# Perceptions of Obstetrics/Gynecology Surgeons on Non-medically Indicated Cesarean Sections: A Cross-Sectional Study

**DOI:** 10.7759/cureus.44508

**Published:** 2023-09-01

**Authors:** Nasreen G Majeed, Shakhawan A Mustafa, Abdelrahman M Makram, Paxshan A Mohammed, Jeza M Abdul Aziz, Mina M Mansour, Dilsoz M Qadir, Ali T Arif, Maryam B Mahmmod, Mariwan K Rasheed, Nguyen Tien Huy

**Affiliations:** 1 Obstetrics and Gynaecology, Baxshin Research Center, Baxshin Hospital, Sulaymaniyah, IRQ; 2 Health Sciences, Kurdistan Institution for Strategic Studies and Scientific Research (KISSR), Sulaymaniyah, IRQ; 3 School of Public Health, Imperial College London, London, GBR; 4 Biomedical Sciences, Komar University of Science and Technology, Sulaymaniyah, IRQ; 5 Baxshin Research Center, Baxshin Hospital, Sulaymaniyah, IRQ; 6 Faculty of Medicine, South Valley University, Qena, EGY; 7 Obstetrics and Gynecology, University of Sulaimani, Sulaymaniyah, IRQ; 8 Medical Laboratory of Science, University of Human Development, Sulaymaniyah, IRQ; 9 School of Tropical Medicine and Global Health, Nagasaki University, Nagasaki, JPN

**Keywords:** non-medically indicated cesarean section, obstetrics/gynecology, cross-sectional study, elective cesarean section, cesarean section on request

## Abstract

Background: Numerous factors can influence decisions regarding the type of delivery of human babies. There is an increasing demand for non-medically indicated cesarean sections (CS) (non-miCS) or CS on request (CSor). Therefore, this survey study aimed to identify the factors that may foster the decision of CS among obstetricians.

Methods: After the sample size calculation returned with 132 needed participants, confidence surveys were sent electronically or disseminated in paper form to nearly all obstetricians (around 200) in the province between mid-August 2021 and mid-February 2022. After signing the consent form, obstetricians were able to provide responses to the four sections of the questionnaire. Data from the copies of the paper were entered into Excel by a local data collector. The data analysis was done using Statistical Product and Service Solutions (SPSS) (IBM SPSS Statistics for Windows, Armonk, NY) and followed the following sequence: summary statistics were done first; then the groups (for and against non-miCS) were compared using analysis of variance (ANOVA); and, finally, regression models were conducted to determine the factors that may affect the favorability of doing non-miCS.

Results: A total of 104 obstetricians responded to the survey. Approximately 62.5% of them performed CSor for women who requested it. In addition, more than half (57.7%) agreed that all women had the right and autonomy to choose their mode of delivery. Most providers (65.4) agreed that fear of vaginal delivery (VD) and a bad experience with it are rational reasons for performing a CSor. Unfortunately, some obstetricians (18.3%) faced lawsuits when they refused to perform CSor. As for the factors that may influence the acceptance of obstetricians to non-miCS, it was found that obstetricians who are unsure or refuse to answer (OR=4.30, 95%-CI 1.25-16.29, p=0.025), along with people who do not always perform CSor (OR=4.33, 95%-CI 1.59-12.50, p=0.005) or even refuse it (3.54, 95%-CI 1.05-12.96, p=0.046), are more likely to agree that women have the right to request CSor.

Conclusion: The surge in CSor rates was mostly correlated with an attempt to escape the fear of VD. However, given the wide discrepancies in obstetricians’ opinions in this survey, we cannot draw firm conclusions about the reasons behind this phenomenon. It is also important to explore possible ways to address the problem, such as through litigation with providers who refuse to perform a CSor and through economic reform to protect women from money-grubbing obstetricians.

## Introduction

The increasing rate of cesarean section (CS) during the last two decades has become a significant issue for the health system worldwide [1]. Surgical operations are associated with a significant increase in infant and maternal morbidity and mortality, particularly in developing countries. Iraq is one of the countries with a higher CS prevalence than recommended [2]. The overall CS rate for all births in Iraq in 2012 was 24.4%, with the highest prevalence in the Sulaymaniyah governorate [3]. In 2018, CS rates increased remarkably compared to the rates of the 2011 survey [4]. The predominant factor behind this seems to be the trend toward non-medically indicated CS, which is also called “on the patient’s request” [5]. Non-medically indicated CS can be due to various reasons, including fear of perineal tears, obstructed labor, and previous recurrent pregnancy loss [6].

According to the WHO statement in 2021, a CS rate higher than 10% will not lead to a decrease in the rate of maternal or fetal mortality and is associated with increased surgical complications and disabilities, especially when surgery is not performed appropriately [7]. Any CS might result in short-term complications, including bleeding, infection, anesthesia complications, and the requirement for additional analgesics and antibiotics. Long-term complications have also increased as the number of CS has increased, including adhesions, abdominal organ injuries, infertility, and abnormal placentation such as placenta previa and scar ectopic pregnancy. Additionally, newborns delivered through CS are more susceptible to short-term health issues, such as transient tachypnea and necessary admission to neonatal units. Moreover, some forms of hormonal, physical, and altered immune development, such as allergy, atopy, asthma, and reduced diversity of the intestinal gut microbiome, may occur [8-10].

Regardless, the causes of non-medically indicated CS are not well-understood in various places around the world. Discovering the motivation behind such an act will help in planning interventions to tackle a procedure that carries an increased risk to the baby and mother. This study aimed to determine and assess the factors behind the increased tendency toward non-medically indicated CS among obstetricians, to identify the main reasons leading to some obstetricians’ preference for CS on request (CSor), assess the perception of obstetricians toward non-miCS, and report how some obstetricians may deal with certain scenarios of CSor.

## Materials and methods

This survey study was performed in line with the principles indicated in the Consensus-Based Checklist for Reporting of Survey Studies (CROSS) checklist [11].

Study population and sample size calculation

This was a generalized study that did not restrict the sample size. We included currently working obstetricians who were only serving in the Sulaymaniyah governorate or its periphery. There were no limitations on age, years of experience, sex, workplace type (e.g., governmental or private), religion, ethnic background, or any other basic characteristics. However, we excluded obstetricians who were retired, operated outside the bounds of the governorate, and refused to participate. It is noteworthy that no male obstetricians were allowed to practice in our setting. Regardless, the sample size was calculated to determine the number of healthcare providers needed to produce generalizable results.

The sample size calculation was adopted from Pourhoseingholi et al.’s paper [12]: [N=λ2p (1-p)/d2], where N is the sample size, λ is the confidence level (set at 1.96 for 95%), p is the approximate number of obstetricians in the province, and d is the tolerance (set at 0.05). As the approximate number of obstetricians in the province is 200, the required sample size was 132.

Questionnaire design and conduction

The questionnaire was separated into five subcategories: electronic informed consent form, basic demographic information, opinions, surgical expertise in specific scenarios, and opinions on performing CS on request (Csor) of their patients.

The types of questions drafted for this study were based on other relevant obstetrical survey studies [13,14] and included simple (yes/no) responses, open-ended questions, and all-that-apply questions. The questionnaire was drafted in English and translated into local dialects by native English speakers. The translated version was validated by independent local doctors. Finally, forward-backward translation was performed to ensure the proper delivery of the meaning. The questionnaire forms were either provided in person (printed hard copies) or sent via email containing a link to the SurveyMonkey survey. The questionnaires were distributed to obstetricians who performed CS. Informed consent was obtained after explaining the purpose of the study and ensuring that the participants met the inclusion criteria. All the data used were collected between 15/08/2021 and 15/02/2022.

Data analysis

The results from the questionnaires were transferred to a Microsoft Excel spreadsheet. All analyses were performed using Statistical Product and Service Solutions (SPSS) Statistics Software version 22.0 for Windows and Microsoft Excel (IBM SPSS Statistics for Windows, Armonk, NY). Simple frequency tables were used for all variables. For response data from the open-ended questions, the results were presented qualitatively (using thematic analysis).

The comparison was performed between obstetricians who declared that women had the right to request CSor against those who did not approve of that right, using the analysis of variance (ANOVA) test for continuous variables and Fisher’s exact test for categorical variables. The compared variables were age, years of experience, the highest degree achieved, and whether obstetricians would consider having CSor for themselves. The aim of the comparison was to identify any statistically significant differences in the basic characteristics that may influence the participation of obstetricians in the study. Homoscedasticity when performing ANOVA was checked using Bartlett’s test. Statistical significance was set at p<0.05. In addition, to determine which variables affected obstetricians’ agreement for women to have complete legal rights and medical autonomy in choosing to deliver using CSor, we performed simple and multiple logistic regression models. The choice of covariates in multiple regression was determined using stepwise backward elimination, in which the final model should not have a change in the estimates of other covariates of >5%. The final model included age, whether obstetricians considered having CSor for themselves, and whether obstetricians regularly performed CSor. Along with P<0.05, statistical significance was determined if the 95% confidence interval (CI) of the odds ratio (OR) did not cross or include a value of 1.

Ethical considerations

Ethical approval for this study was obtained before data collection from the Ethics Committee on 08/08/2021. The ethical approval identification number was 159. Potential risks were considered and determined to be limited to the minimal burden of time and stress induced by responding to the survey. There was no incentive for participation (financial or otherwise) outside the pursuit of improving healthcare.

Electronic informed consent was obtained from obstetricians. If the obstetrician answered “YES” to the first question on the electronic survey form, they would have automatically agreed to participate and would have begun answering the survey. Using the skip logic survey method, any participant who disagreed with the informed consent question was directed toward the end of the survey. If an obstetrician preferred a hard copy to complete the survey, a signature was obtained. None of the respondents were forced to participate in the survey, and their participation was based on their agreement. All participants were free to decline or withdraw from the study at any time.

All participants’ personal information remained confidential and their responses, once recorded on the data management digital spreadsheet, were destroyed. Regarding electronically collected data, IP address tracking was disabled to disallow any attempt to identify the enrolled obstetricians. All data will be stored for five years after the publication of this manuscript.

## Results

Of the 107 invited obstetricians, 104 (97.2%) participated in the study and electronically signed informed consent forms. Table 1 summarizes the characteristics of the 104 obstetricians enrolled in this study. All the providers surveyed were female with a mean age of 46.76 (range 34-72) and mean years of experience of 13.99 (range 1-40).

**Table 1:** Basic information of the recruited obstetricians was accompanied by a comparison of their characteristics according to their acceptance that women have the right to request CSor. Categorical variables are presented as frequencies and percentages between parentheses. The p-values for continuous variables were obtained using the analysis of variance (ANOVA) test, whereas the p-values for categorical variables were obtained using Fisher’s exact test. Homoskedasticity when performing an ANOVA was checked using Bartlett’s test. Abbreviations: MBChB (Bachelor of Medicine and Bachelor of Surgery); DOG (Diploma in Obstetrics and Gynaecology); HDOG (High Diploma in Obstetrics and Gynaecology ); SD (standard deviation); IQR (interquartile range); VD (vaginal delivery); CS (cesarean section); CSor (CS on request); miCS (medically indicated CS); MoD (mode of delivery).

Characteristics	Total (N=104)	Agree group (N=61)	Disagree group (N=43)	P-value
Age				
Mean (SD)	46.76 (8.45)	46.48 (8.54)	47.16 (8.41)	0.418
Median (IQR)	45.50 (13.80)	45.00 (13.00)	46.00 (14.00)	
Range	34 to 72	24 to 64	31 to 72	
Years of experience				
Mean (SD)	13.99 (8.830)	14.28 (8.35)	13.58 (9.55)	0.765
Median (IQR)	12.50 (14.5)	12.00 (14.00)	13.00 (13.00)	
Range	1 to 40	2 to 30	1 to 40	
Medical degree				
MBChB	7 (6.7%)	1 (1.6%)	6 (14.0%)	0.013
DOG	21 (20.2%)	13 (21.3%)	8 (18.6%)	
HDOG	43 (41.3%)	24 (39.3%)	19 (44.2%)	
Board certified	33 (31.7%)	23 (37.7%)	10 (23.3%)	
Consider having CSor for themselves				
Yes	29 (27.9%)	23 (37.7%)	6 (14.0%)	0.023
No	51 (49.0%)	28 (45.9%)	23 (53.5%)	
Not sure or refused to answer	24 (23.1%)	10 (16.4%)	14 (32.6%)	

**Figure 1:**
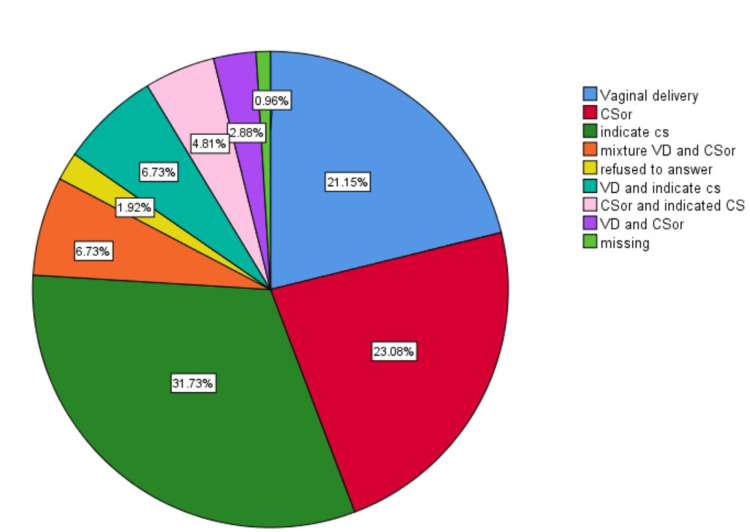
Obstetrician infants' modes of delivery.

In addition to the information provided in the table, it was found that 21.2%, 23.1%, 31.7%, 6.7%, and 4.8% had delivered their own babies only via VD, CSor, medically indicated CS (miCS), a mixture of VD and different modalities of CS (miCS and/or CSor), and a mixture of CSor and miCS, respectively (Figure 1).

**Figure 2:**
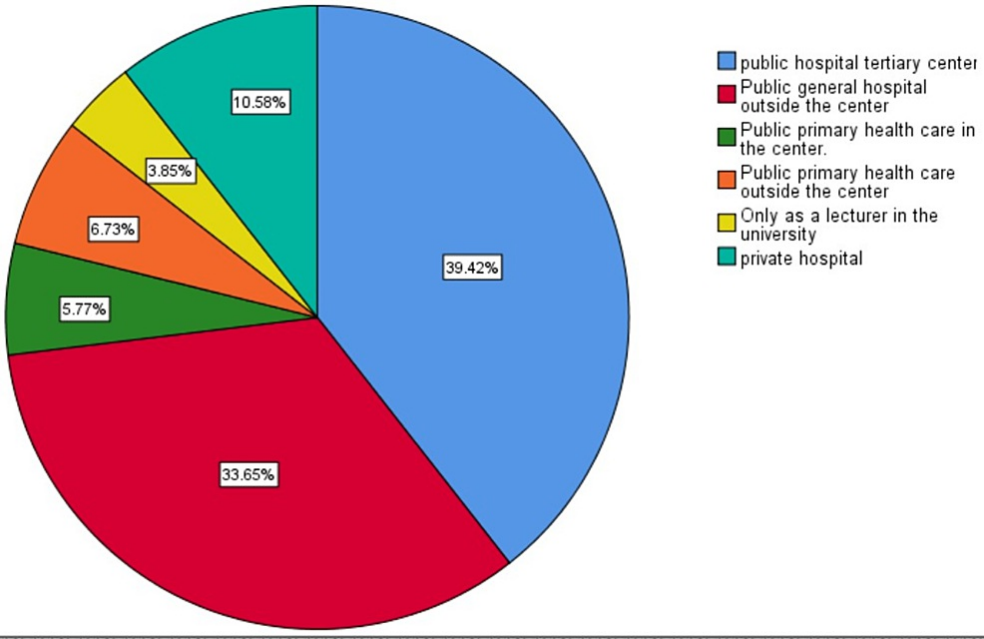
Obstetricians' workplace.

Of the 104 obstetricians enrolled in the study, 39.4% worked in public hospital tertiary centers, 33.7% in public general hospitals outside the center, 14.4% in private hospitals, 6.7% in public primary healthcare in the center, and 5.8% in public primary healthcare outside the center (Figure 2).

Upon asking obstetricians about performing CSor, 62.5% answered yes, 13.9% said no, and 24.0% indicated that they only do it sometimes. Most providers (57.7%) agreed that women had the right to request a CSor, while only 42.3% disagreed. Of the obstetricians, 57.7% believed that all patients should have autonomy in CSor, regardless of age, educational level, or social aspects. Contrastingly, 30.8% believed that any CSor should not be performed, 5.8% wanted to discourage only very young mothers against CSor, 3.8% believed that only people with low education levels should be discouraged against CSor, and 1% said that only the poor should be discouraged against CSor (Table 2).

**Table 2:** Opinions and experience of the recruited obstetricians. Abbreviations: SD (standard deviation); IQR (interquartile range); VD (vaginal delivery); CS (cesarean section); CSor (CS on request); miCS (medically indicated CS); MoD (mode of delivery).

Opinions and experience	Frequency	Percentage
Total	104	100%
Performing CSor		
Yes	65	62.5%
No	14	13.9%
Not always	25	24.0%
Agreeing that women have the right for a CSor		
Agree	60	57.7%
Disagree	44	42.3%
Who should have autonomy for CSor?		
All patients should have autonomy regardless of age, educational level, and any social aspects	60	57.7%
Only very young mothers should be discouraged against CSor	6	5.8%
Only people with low education levels should be discouraged against CSor	4	3.8%
Only the poor should be discouraged against CSor	1	1%
Regardless of any other aspects, any CSor should not be performed	32	30.8%
Missing	1	1%
Which non-medical indications do you consider applying for a CSor?		
Fear of VD		
yes	68	65.4%
No	36	34.6%
Preserve vaginal anatomy		
yes	39	37.5%
no	65	62.5%
Prior bad experience of VD		
yes	68	65.4%
no	36	34.6%
Prior good experience of CS		
yes	9	8.7%
no	95	91.3%
Fear for baby's health		
yes	39	37.5%
no	65	62.5%
Unjustified husband's request		
yes	7	6.7%
no	97	93.3%
Unjustified mother's request		
yes	5	4.8%
no	99	95.2%
Prior bad experience with refusing CSor		
Yes	57	54.8%
No	46	44.2%
Missing	1	1%
Prior litigation for refusing CSor		
Yes	19	18.3%
No	84	80.8%
Missing	1	1%

However, only 27.9% of obstetricians considered having CSor for themselves (Table 1). When asking obstetricians about the non-medical indications for applying CSor, fear of VD and a previous bad experience with VD predominated (65.4%). Other reasons included preservation of vaginal anatomy (37.5%), fear of the baby’s health (37.5%), prior good experience with CS (8.7%), unjustified husbands’ requests (6.7%), and unjustified mothers’ requests (4.8%). Most obstetricians (54.8%) had prior bad experiences with refusing CSor, while only 18.3% had prior litigation when refusing CSor (Table 2). Moreover, using ANOVA, it was found that obstetricians who agreed that women had the right to request CSor were more likely to request CSor for themselves (p=0.023) and perform CSor for others (p=0.002). Their decision is unlikely to be affected by prior experience of refusing CSor (p=0.531) or previous litigation (p=1.000).

To determine how obstetricians deal with different and special situations, they were asked their opinions about specific cases (Table 3).

**Table 3:** Answers to case scenarios provided by recruited obstetricians. Abbreviations: SD (standard deviation); IQR (interquartile range); VD (vaginal delivery); CS (cesarean section); CSor (CS on request); miCS (medically indicated CS); MoD (mode of delivery).

Answers	Frequency	Percentage
Total	104	100.0%
Case 1		
Question 1		
Perform the CS anyway	2	1.9%
Trying to persuade her to VD	90	86.5%
Refuse to perform CS	10	9.6%
Refer to colleague	2	1.9%
Question 2		
She has the autonomy to decide her MoD	75	72.1%
If I refuse, she will go to a colleague	27	26.0%
The absence of motivation will lead to failed VD	35	33.7%
Question 3		
She is very young to make the appropriate decision	33	31.7%
She has no medical indication for CS	71	68.3%
Case 2		
Question 1		
Perform the CS anyway	1	1.0%
Trying to persuade her to VD	78	75.0%
Refuse to perform CS	24	23.1%
Refer to colleague	1	1.0%
Question 2		
She has the autonomy to decide her MoD	74	71.2%
If I refuse, she will go to a colleague	25	24.0%
The absence of motivation will lead to failed VD	32	30.8%
Question 3		
She is old enough, but her past uncomplicated VD should encourage her against CSor	73	70.2%
She has no medical indication for CS	29	27.9%
Missing	2	1.9%
Case 3		
Question 1		
Perform the CS anyway	8	7.7%
Trying to persuade her to VD	89	85.6%
Refuse to perform CS	5	4.8%
Refer to colleague	1	1.0%
Missing	1	1.0%
Question 2		
She has the autonomy to decide her MoD	76	73.1%
If I refuse, she will go to a colleague	29	27.9%
The absence of motivation will lead to failed VD	35	33.7%
Question 3		
She is well-educated and should know that CSor can lead to more complications than VD	67	64.4%
She has no medical indication for CS	37	35.6%
Case 4		
Question 1		
I will recommend CS	2	1.9%
I will tell her she has no medical indication for CS	69	66.3%
I will explain all possibilities but leave the decision to her	31	29.8%
Missing	2	1.9%
Question 2		
I am afraid of medical misconduct and possible litigation	46	44.2%
I am not sure she will have a proper intrapartum assessment	57	54.8%
Missing	1	1.0%
Case 5		
I will tell her something to cover up the senior doctor to avoid a bad reputation	19	18.3%
I will tell her she has no medical indication for CS	49	47.1%
I will tell her there would be a chance of failure to progress	34	32.7%
Missing	2	1.9%
Case 6		
Question 1		
I will perform CS as I do not trust junior doctors and midwives when dealing with shoulder dystocia	55	52.9%
I am afraid of medical misconduct and possible litigation	47	45.2%
Missing	2	1.9%
Case 7		
Question 1		
I will transfer her to a private hospital and continue delivery myself	52	50.0%
I will transfer her to a private hospital and delegate to a colleague	14	13.5%
I will refuse to transfer her to a private hospital	34	32.7%
Missing	4	3.8%
Question 2		
Labor wards in private hospitals do not have the facilities to deal with such cases	69	66.3%
The price is not deserved for such a hard work	30	28.6%
Missing	5	4.8%

The first case was an adolescent primigravida lady at 37 weeks of gestation, with a cephalic presentation, no antepartum problems, and average height of the mother, and the baby was of average size on ultrasound and examination. She is asking for CS. Most obstetricians (86.5%) answered that they should try to persuade her about VD, but they (72.1%) also thought that she had the autonomy to decide her mode of delivery (MoD) and indicated that if they refused, she would go to another surgeon (26%) or inevitably have a failed VD because of the lack of motivation (33.7%). This was despite the belief that she had no medical indications for CS (68.3%).

The second case was of a 28-year-old pregnant woman, gravida 4 para 3, low-risk pregnancy, at 38 weeks of gestation, with a cephalic presentation, and had three uncomplicated VDs before. The last child was one and a half years old. She requested CS because she could not tolerate the pain. Similarly, 75% indicated that they would try to persuade her about VD, and 71.2% said that she had the autonomy to decide her MoD (71.2%). Unlike the first case, 70.2% thought that she was old enough to make such a decision, but her past uncomplicated VD encouraged her to oppose CSor. Nevertheless, some still believe that, if they refuse to provide their requested care, they will go to another doctor (24%) or have a failed VD (30.8%).

The third case involved a 29-year-old pregnant woman, primigravida, well-educated, and planning to have only two children. She asked for non-miCS. The majority (85.6%) said that they would try to persuade her about VD. However, if that fails, they also believe that she is well-educated (64.4%) and should know that CSor can lead to more complications than VD.

In the fourth case, the patient wanted a second opinion regarding a low-risk pregnancy. The sonographer told her that “the cord is around the neck, and the baby has a risk in case of VD.” Surprisingly, only 66.3% would tell her that she has no medical indication for CS, whereas 54.8% said that they would not be sure that she would have a proper intrapartum assessment; therefore, they would perform CS for her. This is an important point to consider, as some informally trained specialists (e.g., sonographers and midwives) may influence the decision to perform VD/CS.

In the fifth case, a young primigravida with low risk, gestational age of ≥ 37 weeks, average baby size, cephalic presentation, no cervical dilatation, and at station 3, was told by another senior colleague that the head was high, and she could not deliver vaginally. Still, only (47.1%) answered that they would tell her that she had no medical indications for CS.

The sixth case was a low-risk multiparous woman at 38 weeks of gestation. On clinical examination and ultrasound, the baby weighed approximately 4 kg. She was told that the baby was big and that there was a chance for shoulder dystocia. The patient preferred VD but was very anxious about the baby. Specifically, 52.9% answered that they would perform CS, and 45.2% were afraid of medical misconduct and possible litigation.

The final case was a 22-year-old primigravida, low risk, at 40+4 weeks of gestation, cephalic presentation, average size of the baby with no cervical dilatation, and a Bishop score of 2. It does not matter for her what her MoD is, but she refuses to have labor at a public hospital. On her request, 50% answered that they would transfer her to a private hospital and continue delivery by themselves (13.5), and 66.3% thought that labor wards in private hospitals do not have the facilities to deal with such cases.

As for the factors that may influence whether obstetricians agree to have their complete legal rights and medical autonomy in choosing whether to deliver using CSor, it was found that obstetricians who are unsure or refuse to answer (OR=4.30, 95%CI 1.25-16.29, p=0.025), along with people who do not always perform CSor (OR=4.33, 95%CI 1.59-12.50, p=0.005) or even refuse it (OR = 3.54, 95%CI 1.05-12.96, p=0.046), are more likely to agree that women have the right to request CSor (Table 4).

**Table 4:** Simple and multiple logistic regression models were used to predict the agreement of women with the right to request CSor. *Indicates statistical significance at p<0.05. † Board certification (n=33) was used as the reference value instead of MBChB (n=7) because the comparatively lower number of people with MBChB led to an error during the model calculation.

	Univariable logistic regression			Multiple logistic regression		
Variables	OR	95% CI	P-value	OR	95% CI	P-value
Age in years	1.02	0.98-1.06	0.416	Excluded		
Years of experience	0.99	0.95-1.04	0.763	0.98	0.93-1.03	0.376
Medical degree				Excluded		
MBChB or DOG	2.30	0.82-6.73	0.120	–	–	–
HDOG	1.82	0.71-4.86	0.219	–	–	–
Board certified	Reference^†^			–	–	–
Consider having CSor for themselves						
Yes	Reference			Reference		
No	2.91	1.06-8.97	0.047*	2.41	0.81-7.93	0.126
Not sure or refuse to answer	5.13	1.59-18.42	0.008*	4.30	1.25-16.29	0.025*
Performing CSor						
Yes	Reference			Reference		
No	3.83	1.21-12.96	0.024*	3.54	1.05-12.96	0.046*
Not always	4.54	1.74-12.57	0.003*	4.33	1.59-12.50	0.005*
Prior bad experience with refusing CSor				Excluded		
Yes	Reference			–	–	–
No	1.28	0.59-2.83	0.531	–	–	–
Prior litigation for refusing CSor				Excluded		
Yes	Reference			–	–	–
No	0.73	0.35-2.72	0.941	–	–	–

More than half of the surveyed obstetricians (61.6%) believed that increasing the cost of CSor was an effective way to reduce its prevalence. On the other hand, some (56.6%) thought that increasing the cost would motivate malpractice and have negative effects on the patient-doctor relationship and the patient’s view of the doctors (55.6%). Ultimately, 51.5% of obstetricians said that they preferred to have CS in the private section rather than VD, and 47.5% thought that CS was safer for the baby than VD (Table 5).

**Table 5:** Final opinion of the recruited obstetricians about the subject of CSor. Abbreviations: SD (standard deviation); IQR (interquartile range); VD (vaginal delivery); CS (cesarean section); CSor (CS on request); miCS (medically indicated CS); MoD (mode of delivery).

Opinions	Frequency	Percentage
Total	99	90.8%
Increasing the cost of CS on request is a good idea to decrease the prevalence of CSor		
Yes	61	61.6%
No	38	38.4%
Missing	0	0%
Increasing the cost of CS will motivate malpractice doctors to do more CSor		
Yes	56	56.6%
No	42	42.4%
Missing	1	1.0%
Increasing the cost of CS will have negative effects on the patient-doctor relationship and the patient’s view of the doctors		
Yes	55	55.6%
No	44	44.4%
Missing	0	0%
I prefer to have CS in the private section rather than VD		
Yes	51	51.5%
No	48	48.5%
Missing	0	0%
I think CS is safer for the baby than VD		
Yes	47	47.5%
No	52	52.5%
Missing	0	0%

## Discussion

This study aimed to investigate the opinions behind the increased rates of non-miCS in the Sulaymaniyah governorate according to physicians. Of the 104 obstetricians who participated in the study, only 30.8% believed that non-miCS should not be performed, while 57.7% agreed that women should retain autonomy in their choice of MoD. Since all obstetricians are female, the qualitative reporting of their experience in this study was surprising, as a quarter of them would prefer CS over VD for themselves. The most frequently cited reasons were fear of pain during VD, fear of episiotomy, previous complicated VD, or previous uncomplicated CS. When asked about specific situations, one in four surgeons feared losing the "client" to a colleague if they refused to perform non-miCS on the pregnant woman. It is also noticed that some obstetricians would prefer doing a non-miCS for the very young or the not-very-well-educated because they might think that they do not know what is best for them, even though most of them would always persuade any woman asking for non-miCS to have a normal VD (75-86%). However, only one-third of respondents believe that patients have the autonomy to choose their MoD (71-72%). Physicians from the United States [15] and China [16] have also indicated that the decision to have a cesarean delivery is the sole responsibility of a pregnant woman and that she should always retain full autonomy in her decision. In a study by Michalik et al., it was found that 96.2% of midwifery students and 68% of medical students thought that delivery in labor was the safer route in low-risk labor [17]. These findings are consistent with the results of the study by Monari et al. in which they found that midwives were more suspicious of the benefits that a CSor may have for pregnant women than obstetricians (p=0.02) [18]. This may be in contrast to the results of our study, as less than one-third of obstetricians considered CSors themselves. However, it is generally recommended that the patient be informed of all possible maternal and neonatal consequences before deciding whether to undergo a CS [19]. Proper consideration and presentation of risks and benefits can significantly influence women’s decisions.

This study also reports a new finding that has received little attention in the literature. We found that there are differences between the education/experience levels of obstetricians who agree and obstetricians who disagree that women have the full legal right and medical autonomy to decide whether to deliver with CSor (p=0.013). Other differences were found, namely, obstetricians considering CSor for themselves were more likely to agree that women have sole decisions and control over the mode of delivery (p=0.023). Surprisingly, obstetricians who did not always or did not perform CSor were more likely to accept the idea of women's sole autonomy in choosing CSor. Similar studies from other countries have confirmed these findings by concluding that senior obstetricians tend to perform more CSor than relatively less experienced surgeons [20,21].

It is noteworthy that many obstetricians are afraid of losing the "client" to another colleague or fear of poor management in public hospitals by a resident or midwife. Other studies have, for example, found that ill-prepared theatres, senior obstetricians, residents, nurses, and midwives in emergency situations are big drivers of CSor [22-25]. Furthermore, the private sector reimburses CS far more than VD in many instances. This has made some obstetricians convince women that CSor is safer or inadvertently indicated for them [25-27]. Therefore, it can be assumed that the economic aspects of CS (scheduled, higher cost for the surgeon, and less working time) are detrimental factors in the eyes of a surgeon. This should be done within the framework of a system that focuses on providing the best possible care to the mother without relying too much on the private sector, which seems to focus more on fiscal gain.

Moreover, it is important to mention how other medical professionals can influence the decision to have CSor. For example, midwives are sometimes bothered by the inadvertent CSor decision employed by the obstetrician [28]. The same problem is regenerated for early career residents as they are typically less involved in the decision-making process [25]. Other people who may influence the high CSor rate is the fear of anxiety, mainly manifested by psychiatrists co-managing a fearful patient [29,30]. However, there is a scarcity of research on the true influence of other medical professionals in CSor decisions. Further research is required in this area.

This study had several limitations. One limitation is that there were only female obstetrician attendants, which may limit the different opinions obtained from the opposite sex. Nevertheless, this is the first study in the Middle East to examine obstetricians' attitudes and opinions regarding non-miCS. However, the study may have some internal validity issues, and the generalizability of the conclusions drawn in this study is still at risk. Positive or negative corroboration of these findings by other researchers from the same or other countries is strongly recommended to learn more about obstetricians' behavior in this area.

## Conclusions

This study aimed to investigate the reasons for the increase in CSor rates in the Sulaymaniyah governorate through interviews with obstetricians. We found that it was mostly related to trying to avoid the fear of VD to achieve a peaceful postoperative course. However, the wide discrepancies between the opinions of obstetricians in this survey were unable to draw definite conclusions regarding the true reasons for this phenomenon. It may seem illogical that people would want a "safer" delivery when they and their babies experience a worrisome postnatal course. Also surprising was the finding that obstetricians themselves are more reluctant to advise CSor in the absence of a clear high-risk pregnancy profile or a clear CS indication. Addressing economics in conjunction with eliminating knowledge gaps and misconceptions about obstetricians may help solve this problem.
